# Mechanisms of Pesticide Toxicity in Fish: Insights Into the Ameliorative Role of Plant-Derived Compounds—A Review

**DOI:** 10.1155/anu/5328773

**Published:** 2025-07-09

**Authors:** Sayyed Ali Moezzi, Sudabe Ramezani, Kiadokht Rezaei, Arash Javanshir Khoei

**Affiliations:** ^1^Department of Fisheries, Faculty of Natural Resources, University of Tehran, Karaj, Iran; ^2^Department of Aquatic Animal Health, Faculty of Veterinary Medicine, University of Tehran, Tehran, Iran

**Keywords:** aquaculture, fish, immunity, oxidative stress, pesticide, phytochemicals, stress amelioration

## Abstract

Pesticide contamination in aquatic environments poses severe risks to fish health, causing oxidative stress, immunosuppression, endocrine disruption, and neurotoxicity. These effects result from the accumulation of reactive oxygen species (ROSs), enzyme inhibition, and damage to physiological systems. Plant-derived compounds and phytochemicals, such as polyphenols, flavonoids, alkaloids, saponins, and terpenoids, offer a sustainable strategy to mitigate these toxic effects due to their antioxidative, anti-inflammatory, immunomodulatory, and detoxifying properties. Phytochemicals protect fish by scavenging ROS, upregulating antioxidant enzymes (i.e., superoxide dismutase, catalase, and glutathione peroxidase), and enhancing detoxification pathways (i.e., cytochrome P450 enzymes and glutathione-S-transferase). They also reduce inflammation by inhibiting pro-inflammatory cytokines and NF-κB signaling and restore immune function by improving phagocytic activity and lymphocyte proliferation. Additionally, phytochemicals counter endocrine disruption and neurotoxicity by stabilizing hormone levels and preventing the accumulation of acetylcholine in neural pathways. Incorporating plant-derived compounds into fish diets can reduce oxidative damage, strengthen immune responses, and enhance fish resilience to pesticide exposure. This review emphasizes the potential of phytochemicals to promote safer, more sustainable aquaculture practices. Further research on dosage and application methods could lead to significant advancements in aquatic toxicology and fish health management.

## 1. Introduction

Aquatic ecosystems are increasingly threatened by agricultural activities, particularly the widespread use of pesticides [1, 2]. Although the use of pesticides such as organophosphates, carbamates, pyrethroids, and neonicotinoids is necessary to eliminate pests in agriculture, they can threaten the health of nontarget organisms including fish, when they enter water bodies through runoff, leaching, or atmospheric deposition [3–8]. These toxic chemicals can accumulate in fish tissues, leading to a range of deleterious effects, such as oxidative stress, immunosuppression, endocrine disruption, and neurotoxicity, all of which compromise fish health, reproduction, and survival [9–11]. Given the growing environmental concerns associated with pesticide contamination, it is crucial to develop strategies to mitigate their harmful effects on aquatic life. In recent years, plant-derived compounds and phytochemicals have gained considerable attention as natural, environmentally friendly alternatives to chemical detoxifiers [12]. Phytochemicals include a wide range of compounds such as polyphenols, flavonoids, alkaloids, saponins, and terpenoids that have diverse therapeutic functions, including antioxidant, anti-inflammatory and immunomodulatory properties [13–15]. These compounds are widely found in medicinal plants, fruits, vegetables, and herbs, and they have been used in traditional medicine for centuries to treat various diseases [16]. Their potential application in aquaculture to counteract the toxic effects of pesticides has been explored in numerous scientific studies [17–22]. One of the key mechanisms by which plant-derived compounds protect fish from pesticide toxicity is through their ability to combat oxidative stress [23]. Pesticides are known to generate reactive oxygen species (ROSs), which cause oxidative damage to cellular components such as lipids, proteins, and DNA [24]. This leads to lipid peroxidation, protein denaturation, and genetic mutations, all of which impair fish health. Phytochemicals act as potent antioxidants, scavenging free radicals and enhancing the activity of endogenous antioxidant enzymes such as superoxide dismutase (SOD), catalase (CAT), and GPx, thereby mitigating oxidative damage and restoring cellular homeostasis [25].

In addition to their antioxidant properties, plant-derived compounds play a critical role in modulating the immune response in fish [26]. Pesticides are known to suppress both innate and adaptive immunity, reducing the effectiveness of the fish's defense mechanisms against pathogens [9, 27]. Phytochemicals have been shown to enhance the activity of immune cells such as macrophages and lymphocytes, boost antibody production, and stimulate the production of cytokines that regulate the immune response [13, 23]. This immunomodulatory effect not only helps fish fight off infections but also protects them from the immune suppression caused by pesticide exposure.

Another important mechanism by which phytochemicals ameliorate pesticide toxicity is through the activation of detoxification pathways. Pesticides are metabolized in fish through a series of enzymatic reactions, primarily mediated by cytochrome P450 (CYP450) enzymes [12]. These enzymes convert pesticides into less toxic metabolites that can be excreted from the body. However, prolonged pesticide exposure can overwhelm these detoxification pathways, leading to the accumulation of toxic metabolites. Phytochemicals have been shown to induce the expression of detoxification enzymes, including CYP450 and glutathione-S-transferase (GST), which facilitates the biotransformation and elimination of pesticides from the fish's system [28–30]. Although the effects of pesticides on various aspects of fish behavior and physiology have reviewed in many studies [31–33], ongoing toxicological research on fish continues to yield new findings, highlighting the need for updated and in-depth studies in this field. Additionally, new researches have increasingly focused on natural solutions to mitigate the harmful impacts of pesticides, a topic that has received relatively little attention in the context of aquaculture.

Given the growing body of evidence supporting the protective effects of plant-derived compounds and phytochemicals against pesticide-induced toxicity, their application in aquaculture offers a promising and sustainable solution to improving fish health and mitigating the adverse effects of environmental pollutants. This review aims to provide an in-depth analysis of the mechanisms by which phytochemicals exert their protective effects on fish, including their roles in antioxidant defense, immune modulation, and detoxification. By compiling and synthesizing findings from recent scientific studies, this review highlights the importance of integrating phytochemicals into fish diets to counteract the harmful effects of pesticides, ensuring both the health of aquatic species and the sustainability of aquaculture operations. To develop this review, we conducted a comprehensive literature search to identify relevant studies addressing pesticide toxicity mechanisms and the ameliorative role of plant-derived compounds. The literature was gathered from peer-reviewed journals, using scientific databases such as PubMed, Scopus, and Web of Science. By systematically analyzing this body of literature, we aimed to highlight knowledge gaps, synthesize existing findings, and propose directions for future research in the field of aquatic toxicology and fish health management.

## 2. Toxic Effects of Pesticides in Fish

**Figure 1:**
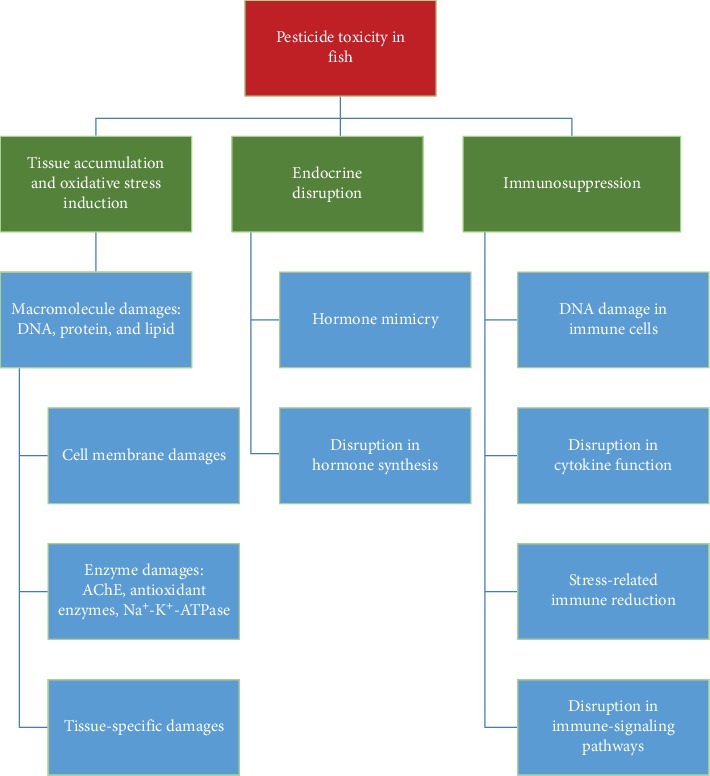
Diagram showing the effects of pesticides in fish.

Pesticides exert a variety of toxic effects on fish (Figure 1), primarily through oxidative stress, neurotoxicity, endocrine disruption, and immunosuppression. These effects are mediated through complex biochemical pathways, ultimately leading to reduced growth, reproductive failure, and increased mortality in fish populations.

### 2.1. Oxidative Stress

Pesticides induce the production of ROS and reactive nitrogen species (RNSs), causing oxidative damage to lipids, proteins, and DNA [24]. This imbalance may overwhelm the fish's antioxidant defenses, leading to lipid peroxidation, protein denaturation, and DNA fragmentation. Pesticides disrupt the mitochondrial respiratory chain and electron transport system [34], generating ROS like superoxide anion, hydroxyl radical, and hydrogen peroxide, which damage cellular components if not neutralized by antioxidants [35]. Pesticide-induced oxidative stress mostly affects fish in different ways as follows:

#### 2.1.1. Lipid, Protein, DNA Oxidation, and Tissue-Specific Disorders

Pesticides generated ROS triggers lipid peroxidation, where ROS attack polyunsaturated fatty acids (PUFAs) in cell membranes, forming lipid radicals and malondialdehyde (MDA), a marker of oxidative damage [31]. This disrupts membrane integrity, increases permeability, and affects ion gradients [36]. The end products of lipid peroxidation, such as MDA and 4-hydroxynonenal (4-HNE) [37], impair proteins and DNA, causing dysfunction. Elevated MDA levels in fish exposed to pesticides indicate significant cellular damage, leading to disrupted cellular signaling and potential cell death, particularly in high-metabolism tissues like the liver and gills [31, 38].

Pesticides also induce protein oxidation [39 ], impairing enzymes like acetylcholinesterase (AChE) [40, 41], which is vital for neurotransmission [42]. This results in neuromuscular disruptions and reduces detoxification capacity by affecting enzymes like GST and SOD [43]. Additionally, ROS causes oxidative DNA damage [44, 45], leading to mutations and potentially carcinogenesis [46–48]. 8-hydroxy-2'-deoxyguanosine (8-OHdG) is a widely used biomarker for oxidative DNA damage, and its levels are significantly elevated in fish exposed to pesticides [47, 49]. The accumulation of DNA damage can negatively affect reproduction and population dynamics, reducing fertility and causing developmental anomalies [45, 50]. Overall, oxidative damage to proteins and lipids can impair the function of a wide range of enzymes and disrupt the cellular membrane in tissues, leading to dysfunction in these tissues. The liver, gills, kidneys, and brain are among the important tissues that can be damaged due to ROS-induced oxidative stress.

The liver plays a central role in detoxifying and biotransforming pesticides in fish, making it highly susceptible to oxidative stress caused by pesticide exposure [31, 51–56]. Elevated levels of MDA, a byproduct of lipid peroxidation, are commonly observed in pesticide-exposed fish, indicating significant oxidative damage [31, 38, 57, 58]. Oxidative stress can disrupt the activity of key enzymes involved in detoxification and antioxidant defense, such as cytochrome P450 [59, 60], CAT, SOD, GPx [31, 61, 62] and nonenzymatic antioxidants like glutathione (GSH), vitamins E and C [63–68]. DNA damage, including strand breaks and base modifications, may occur due to ROS, leading to mutations or apoptosis [69–72]. In addition, the accumulation of ROS can lead to histopathological changes, including hepatocyte vacuolization, necrosis, fatty degeneration, and fibrosis [32, 73–77], ultimately impairing the liver's ability to maintain normal metabolic functions such as protein synthesis and detoxification. These effects can result in reduced liver function and regeneration, further impacting fish health.

Fish gills, essential for gas exchange, osmoregulation, and waste excretion [78], are directly exposed to waterborne pesticides [79]. ROS generated upon oxidative stress damages cell membranes in gill tissue, disrupting structural integrity, ion gradients, gas exchange, and osmoregulation [38, 77, 80, 81]. Elevated MDA levels in pesticide-exposed gills indicate significant oxidative damage [82–84]. Also, ROS-induced protein oxidation [85] disrupts SOD, CAT, and GPx enzymes [86–88] and also alters the structure and function of Na^+^/K^+^-ATPase, a key enzyme for maintaining ion gradients and osmoregulation [89, 90] by oxidizing sulfhydryl groups of the enzyme [91]. A decline in Na^+^/K^+^-ATPase activity has been observed in fish species exposed to pesticides such as chlorpyrifos, cypermethrin, fenitrothion, oxadiazon, and diazinon, resulting in disturbances in sodium and potassium homeostasis [89, 92–96].

Pesticide-induced oxidative stress also causes structural changes in the gill epithelium, including chloride cells, hyperplasia, edema, lamellar fusion, and necrosis [97–99], impairing oxygen uptake, carbon dioxide excretion, and ammonia elimination, ultimately reducing respiratory efficiency. Affected fish may exhibit gasping, lethargy, and decreased swimming performance, indicating impaired respiratory function. Chloride cells, critical for osmoregulation, are also damaged by pesticide toxicity, further affecting ion transport mechanisms [100]. However, an increase in the proliferation of chloride cells was also reported after exposure to diazinon [101, 102] due to increases in pesticide-induced cortisol, a main hormone involved in fish osmoregulation [103]. Hajirezaee et al. [102] observed a decrease in a number of chloride cells after long-term (12 days) exposure of the juveniles of the persian sturgeon, *Acipencer persicus* to 0.18 mg/L diazinon, while these components increased in response to a short-term (96 hr) exposure. Therefore, it seems that the effects of a pesticide on the number and structure of gill chloride cells depend on the exposure dose, duration of exposure, the type of pesticide, fish species, and the test conditions.

Pesticide-induced oxidative stress also affects the fish kidney, a crucial role in osmoregulation, waste excretion, and homeostasis due to its filtration and detoxification functions [78, 104]. The lipid and protein oxidation induced by ROS levels damages Na^+^/K^+^-ATPase enzyme [31, 105] and cell membranes, increasing permeability, and disrupting electrolyte and fluid regulation in the kidney [31, 106]. Pesticide-induced histopathological changes, such as tubular degeneration, glomerular damage, and inflammation, impair kidney filtration, and toxin excretion [9, 31, 107]. Prolonged exposure to pesticides can result in chronic kidney damage, metabolic waste buildup, and compromised fish survival.

The fish brain, crucial for regulating behavior, movement, sensory processing, and endocrine control, is highly vulnerable to ROS generated by oxidative stress due to its lipid-rich composition and metabolic activity [108]. ROS-induced oxidation of lipids and proteins in neuronal membranes may disrupt the structural integrity, fluidity, and permeability of brain cells [105]. Also, the oxidation of proteins can disrupt neurotransmitter systems [109, 110], by inhibiting AChE, leading to acetylcholine accumulation [111]. ROS also can reduce ATP production in mitochondria [112] and triggers apoptosis in neurons, further impairing brain function [34]. The blood–brain barrier (BBB) is a selective barrier that protects the brain from toxins and maintains a stable internal environment for neuronal function [113]. Damage to the BBB allows pesticides and ROS to infiltrate brain tissue, exacerbating oxidative stress and neuronal damage [114–116]. Eventually, the ROS-induced histopathological changes in brain, such as necrosis, vacuolization, and cerebral edema, impair brain functions like learning, memory, and behavior, leading to neurobehavioral disorders and compromised fish survival [117–121].

#### 2.1.2. Engaging Enzymatic and Nonenzymatic Antioxidant System

To counterbalance the ROS produced under normal and stress conditions, fish possess a complex antioxidant defense system composed of enzymatic and nonenzymatic antioxidants [122]. The major antioxidant enzymes include SOD, CAT, and GPx. Also, nonenzymatic antioxidants such as vitamins C and E, glutathione, and selenium also play crucial roles in neutralizing ROS [57, 123]. However, pesticide exposure may overwhelm these defense systems by increasing ROS production beyond the capacity of these antioxidants, leading to oxidative stress. Pesticides may also downregulate the expression and activity of these enzymes, further impairing the fish's ability to cope with oxidative stress [65–68]. Studies have shown that fish exposed to pesticides experience a significant decline in antioxidant enzyme activities, coupled with the depletion of nonenzymatic antioxidants [124, 125]. This decline in antioxidant capacity exacerbates oxidative damage, contributing to the overall toxicity of pesticides. However, an increase in the activity of antioxidant enzymes has been also observed following exposure to pesticides in fish, which could be a defense response to cope with pesticide-induced oxidative stress [126–128]. This kind of response may be an initial reaction, and we may see a reduction in the enzymatic antioxidant responses depending on the duration of exposure, exposure dosage, target tissue, and the type of pesticide [41, 126].

The primary sources of ROSs in fish exposed to pesticides include interference with mitochondrial electron transport, leading to electron leakage and the formation of superoxide radicals. Pesticides are also metabolized by phase I detoxification enzymes, particularly cytochrome P450, resulting in the production of reactive intermediates, including ROS, which contribute to oxidative stress. Additionally, lipid peroxidation occurs, involving the breakdown of PUFAs into smaller components such as aldehydes, further exacerbating oxidative damage [129, 130].

### 2.2. Endocrine Disruption

Endocrine disruption refers to the interference of chemicals, known as endocrine disruptors, with the normal functioning of the endocrine system. These chemicals can alter hormone production, signaling, or action, leading to adverse health effects in humans and animals [131]. One of the key impacts of pesticide exposure is endocrine disruption, which interferes with the normal functioning of the endocrine system, leading to reproductive impairment, developmental abnormalities, and behavioral changes [132]. Endocrine-disrupting pesticides can mimic, block, or interfere with the normal hormonal signals that regulate physiological processes in fish, leading to imbalances in hormone levels and signaling pathways [133, 134]. One of the primary mechanisms by which pesticides induce endocrine disruption is through hormone mimicry, where pesticide compounds structurally resemble natural hormones [135]. These xenoestrogens, xenoandrogens, and xenoprogestins which are classified as xenobiotics, can bind to hormone receptors, such as estrogen receptors (ERs), androgen receptors (ARs), or progesterone receptors (PRs), leading to inappropriate activation or inhibition of hormonal pathways [133, 136]. Xenobiotics are chemical substances that are foreign to a living organism, including drugs, environmental pollutants, and synthetic compounds. They are not naturally produced or expected to be present in the body and can affect biological processes. For example, the pesticides, dichlorodiphenyltrichloroethane (DDT), and nonylphenol are known to act as estrogenic compounds, binding to ERs and mimicking the effects of natural estrogens like estradiol (E2) [137, 138]. This can lead to an increase in estrogenic activity, even in male fish, causing the feminization of male gonads, impaired sperm production, and altered sexual behaviors. Similarly, vinclozolin, an antiandrogenic pesticide, binds to ARs, blocking the normal action of testosterone and disrupting male reproductive development [139, 140].

Pesticides can also interfere with the synthesis and metabolism of hormones, disrupting their normal production and degradation in fish. For instance, triazine herbicides such as atrazine are known to inhibit the enzyme aromatase, which is responsible for converting androgens (e.g., testosterone) into estrogens (e.g., estradiol) [141, 142]. By inhibiting aromatase, atrazine reduces estrogen production, disrupting the normal hormonal balance and affecting reproductive processes like oocyte development and spawning. Additionally, pesticides can alter the metabolism of hormones by interfering with enzymes involved in their degradation, leading to an accumulation or depletion of critical hormones. For example, in Nile tilapia, *Oreochromis niloticus* and other exposed fishes, endosulfan has been shown to disrupt the metabolism of thyroid hormones by inhibiting enzymes such as iodothyronine deiodinase, which is essential for the conversion of thyroxine (T4) into the more active form triiodothyronine (T3) [143, 144]. This disruption of thyroid hormone metabolism can impair growth, development, and metabolic regulation in fish.

The hypothalamic–pituitary–gonadal (HPG) axis plays a central role in regulating reproduction and sexual development in fish [145]. Pesticides can also disrupt this axis at multiple levels, leading to impaired reproductive function. For example, in *Channa punctatus*, metacid-50 and carbaryl have been shown to alter the release of gonadotropin-releasing hormone (GnRH) from the hypothalamus, which in turn affects the secretion of gonadotropin hormones from the pituitary gland [146]. Such impact on GnRH also observed in Asian stinging catfish, *Heteropneustes fossilis* exposed to chlorpyrifos and herbicide 2,4-Dichlorophenoxyacetic acid [147]. Also, Piazza et al. [146] reported a reduction in GnRH secretion after exposure of the cichlid fish, *Cichlasoma dimerus* to 0.1 μg/L endosulfan. Pesticides can also interfere directly with the gonads, altering steroidogenesis, gametogenesis, and overall reproductive capacity. For instance, exposure to endocrine-disrupting pesticides has been associated with ovarian atrophy, testicular degeneration, and reduced fecundity in fish, all of which are mediated by disruptions in the HPG axis [148].

Thyroid hormones play an essential role in regulating metabolism, growth, and development in fish, particularly during critical life stages like larval development and metamorphosis [149]. Pesticides can disrupt the hypothalamic–pituitary–thyroid (HPT) axis, leading to imbalances in thyroid hormone levels [150]. Polychlorinated biphenyls (PCBs) and organophosphates have been shown to reduce circulating levels of thyroxine (T4) and triiodothyronine (T3), leading to impaired growth, altered metamorphosis, and developmental abnormalities in fish larvae [151–153]. In addition to direct disruption of thyroid hormone levels, pesticides can also alter the expression of thyroid hormone receptors, further impairing thyroid hormone signaling pathways. This can lead to reduced metabolic activity, abnormal growth patterns, and reduced survival rates in fish populations [150, 154].

### 2.3. Immunosuppression

Pesticide exposure disrupts both innate and adaptive immune responses in fish [27]. Fish have several key immune organs, including the thymus, spleen, and head kidney, which are analogous to the mammalian bone marrow and spleen and are essential for generating and regulating immune responses [155]. Pesticides can induce structural and functional changes in these immune organs, impairing their ability to produce immune cells and regulate immune responses.

One of the primary mechanisms by which pesticides suppress the immune system is through the induction of oxidative stress [31]. Oxidative stress has been shown to reduce the production of antioxidant enzymes like SOD and CAT, which are important in protecting immune cells from oxidative damage [156–158]. As a result, fish exposed to pesticides exhibit reduced immune activity, making them more vulnerable to infections. Many studies have investigated the effects of pesticides on immune parameters.  Table 1 presents a representive of these studies. The ROS generated by oxidative stress can damage cellular components, including lipids, proteins, and DNA, leading to apoptosis or dysfunction of cellular components and immune cells such as macrophages, neutrophils, and lymphocytes [45, 168, 169]. The overproduction of ROS also disrupts the function of critical immune molecules, such as cytokines and antibodies [170]. Cytokines are signaling proteins that regulate immune responses, including inflammation, cell proliferation, and differentiation in fish [171]. Pesticides have been shown to interfere with the production and function of cytokines, leading to impaired immune signaling. For example, exposure to pesticides such as dichlorvos and chlorpyrifos has been associated with a decrease in the production of proinflammatory cytokines like tumor necrosis factor-alpha (TNF-*α*), interleukin-1 beta (IL-1β), and interleukin-6 (IL-6), which are critical for initiating and sustaining immune responses against pathogens [170, 172].

Pesticide exposure has been shown to suppress humoral immunity by impairing the differentiation of B cells into plasma cells, which are responsible for antibody secretion [173]. Exposure of the common carp to lufenuron and flonicamide significantly decreased the levels of circulating Ig [174]. Similar results were found in the same fish after exposure to cypermethrin [175]. In line with these studies, decreased levels of Ig have been also observed after exposure to pesticides in other fish species [176, 177]. It is recognized that pesticides can impair the signaling pathways involved in promoting B cell proliferation and differentiation into antibody-producing plasma cells such as nuclear factor kappa B (NF-κB), Janus kinase/signal transducer and activator of transcription (JAK/STAT), and mitogen-activated protein kinase (MAPK) [178].

Role of pesticides as endocrine disruptors is another mechanism impairing cellular immunity in fish [133, 179]. Chronic exposure to endocrine-disrupting pesticides can lead to elevated levels of cortisol, a stress hormone that suppresses immune function. Cortisol has been shown to reduce the proliferation of B lymphocytes and inhibit their differentiation into plasma cells [180]. Pesticides can also cause necrosis, a form of uncontrolled cell death, in B lymphocytes. Necrosis leads to the release of cellular contents into the surrounding tissue, triggering inflammation and further immune dysfunction [178, 181]. Pesticides also affect macrophage and neutrophil function by reducing their ability to phagocytose pathogens and produce RNS and ROS, which are essential for microbial killing [182]. The overall effect of pesticide-induced immunosuppression is an increased susceptibility to diseases and infections in fish.

## 3. Major Classes of Plant-Derived Compounds With Protective Effects

### 3.1. Polyphenols

Polyphenols are a diverse group of plant compounds with multiple phenol units and antioxidant properties. They contain an aromatic ring with hydroxyl groups and are classified into subgroups like flavonoids, phenolic acids, tannins, and lignans [183, 184]. Compounds such as quercetin, resveratrol, and curcumin are known for their antioxidant and immune-boosting effects [185, 186]. Found in fruits, vegetables, tea, and coffee, polyphenols offer significant health benefits in humans and animals. In aquaculture, polyphenols play a crucial role in combating oxidative stress and enhancing fish immune function, making them valuable dietary supplements [187].

### 3.2. Terpenoids

Terpenoids, or isoprenoids, are a diverse group of organic compounds derived from isoprene units (C_5_H_8_), synthesized through various patterns [188, 189]. They are classified by the number of isoprene units they contain: monoterpenoids (C_10_), sesquiterpenoids (C_15_), diterpenoids (C_20_), triterpenoids (C_30_), and tetraterpenoids (C_40_), with polyterpenoids having more than eight units. Found in plants, animals, and microorganisms, terpenoids exhibit a wide range of biological activities, including antioxidant, anti-inflammatory, antimicrobial, antitumor, and cardiovascular-protective effects [105, 190]. Their structural diversity enables them to interact with various molecular targets, making them valuable for treating multiple diseases [191, 192]. Examples include limonene, menthol, β-carotene, and astaxanthin [193].

### 3.3. Alkaloids

Alkaloids are a diverse group of naturally occurring organic compounds, primarily containing nitrogen, which are produced by a variety of plants [194]. They are well-known for their wide range of therapeutic properties, many of which have been utilized in traditional and modern medicine. Analgesic, antimicrobial, anti-inflammatory, anticancer activity of alkaloids have been reported in many studies [195]. Reserpine, codeine, quinine, caffeine, and morphine are well-known examples of alkaloids [196].

### 3.4. Saponins

Saponins are plant glycosides known for forming soap-like foams in water and are found in various plants like legumes and cereals [197]. They consist of a sugar molecule attached to a non-sugar component, either a steroid or triterpenoid [198]. Saponins have diverse therapeutic benefits, including antioxidant, immune-boosting, antimicrobial, anti-inflammatory, anticancer, and hepatoprotective properties [197, 199]. These benefits make them valuable in pharmacology, nutraceuticals, and functional foods. Examples include ginsenosides, dioscin, and soyasaponins [200].

### 3.5. Coumarins

Coumarins are a class of naturally occurring organic compounds belonging to the benzopyrone family, widely distributed in plants. These compounds possess a distinctive vanilla-like aroma and have been used in various traditional medicines for centuries. They are primarily found in plants like tonka beans (*Dipteryx odorata*), sweet clover (*Melilotus officinalis*), and citrus fruits. The biochemical structure of coumarins consists of a benzene ring fused with an α-pyrone ring, forming the characteristic benzopyrone structure [201–203]. Coumarins exhibit a wide range of pharmacological activities including antioxidant, antimicrobial, anticoagulant, neuroprotective and hepatoprotective activities, making them valuable for therapeutic applications [203]. Scopoletin, esculin, coumarin, daphnetin, and fraxetin are coumarins with therapeutic properties [204].

### 3.6. Glucosinolates

Glucosinolates are a class of sulfur-containing compounds found predominantly in cruciferous vegetables such as broccoli, cauliflower, Brussels sprouts, kale, and mustard greens [205]. The general structure of glucosinolates consists of three primary components: A β-D-thioglucose group, A sulfonated oxime group, and A variable side chain. These phytochemicals are responsible for the characteristic pungent aroma and bitter taste of these vegetables [205]. When glucosinolates are hydrolyzed by the enzyme myrosinase (which is released during plant tissue damage or through digestion), they are converted into biologically active compounds such as isothiocyanates, thiocyanates, and indoles, which are responsible for many of the therapeutic properties of glucosinolates [205–207]. Glucosinolates and their hydrolysis products have gained significant attention for their health-promoting properties. These compounds have been studied for their roles in cancer prevention, antimicrobial and antioxidant activities, detoxification, anti-inflammatory and hepatoprotective effects [208]. Sulforaphane, glucoraphanin, sinigrin, and glucobrassicin are well-known examples of glucosinolates with therapeutic properties [207].

### 3.7. Polysaccharides

Polysaccharides are complex carbohydrates composed of long chains of monosaccharide units linked by glycosidic bonds. They are found in a variety of natural sources, including plants, fungi, algae, and bacteria. Polysaccharides and their derivatives exhibit diverse therapeutic properties, making them valuable in both traditional medicine and modern pharmacology [209]. Their biological activities are largely dependent on their structural complexity, molecular weight, degree of branching, and the nature of their monosaccharide units [210]. These compounds have gained attention for their role in immunomodulation, antioxidant activity, anti-inflammatory effects, and potential applications in cancer therapy, among other benefits [210]. Astragalus polysaccharides, carrageenan, inulin hyaluronic acid, chitosan, fucoidan, lentinan and Beta-glucans are well-known examples of polysaccharides with therapeutic properties [211].

## 4. Mechanisms of Ameliorating Pesticide Toxicity by Plant-Derived Compounds

**Figure 2:**
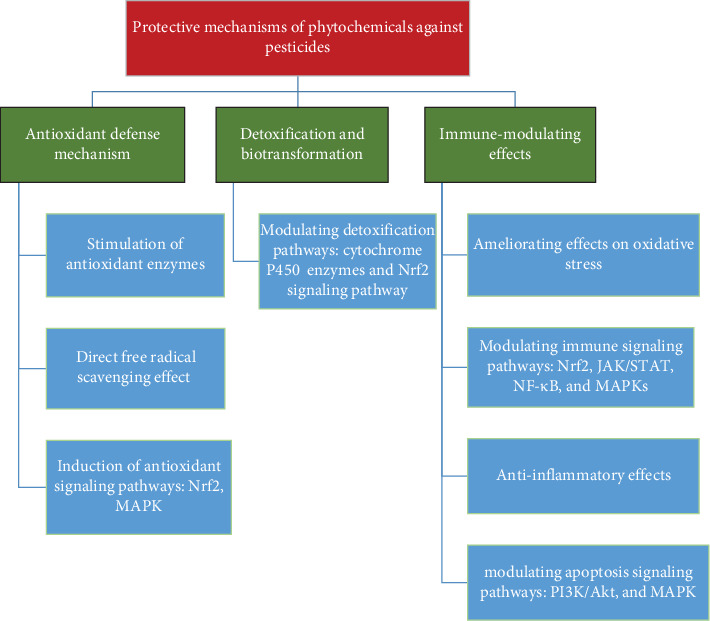
Diagram showing the protective mechanisms of phytochemicals against pesticide toxicity in fish.

As mentioned earlier, pesticide toxicity could be a major concern in aquatic environments, particularly for fish, which are vulnerable to exposure through contaminated water, sediments, and food chains. Plant-derived compounds, also known as phytochemicals, have emerged as potential agents to ameliorate the negative effects of pesticide toxicity in fish (Figure 2). These natural compounds possess antioxidant, anti-inflammatory, and detoxifying properties, which help mitigate the toxic effects of pesticides in aquatic species. Here are some key mechanisms through which plant-derived compounds ameliorate pesticide toxicity in fish.

### 4.1. Antioxidant Defense Mechanism

Pesticide often induce oxidative stress by generating excessive ROS, which disrupts the redox balance, leading to cellular damage, lipid peroxidation, protein oxidation, and DNA fragmentation in fish [212]. To mitigate these toxic effects, the antioxidant properties of phytochemicals have gained considerable attention as natural, eco-friendly alternatives for enhancing fish resilience against pesticide-induced oxidative damage [20, 213–215].

Phytochemicals, including flavonoids, phenolic acids, and terpenoids, possess robust antioxidant capacities that operate through multiple defense mechanisms. These compounds act by either directly scavenging free radicals or enhancing the activity of endogenous antioxidant enzymes, such as SOD, CAT, and GPx [216]. Various studies have shown the role of menthol [217], κ-carrageenan [218], β-glucan, inulin and emodin [219 ],quercetin [220–223], thymol [224–226], curcumin, and resveratrol [227] in enhancing the capacity of antioxidant enzyme defense in fish, in which some showed an ameliorating effect on pesticide-induced oxidative stress [125, 228].

Phytochemicals also upregulate the expression of endogenous antioxidant enzymes via the nuclear factor erythroid 2-related factor 2 (Nrf2) signaling pathway [229–231]. Activation of Nrf2 triggers the transcription of antioxidant response element (ARE)-regulated genes, leading to an increased synthesis of detoxifying and antioxidant proteins, such as GST and NAD (P)H oxidoreductase (NQO1) [232].

Although the modulation of the Nrf2 signaling pathway by phytochemicals has not been investigated with toxicity caused by pesticides, a study by Vineetha et al. [233] showed that *Tinospora cordifolia* can modulate the titanium dioxide nanoparticle-induced toxicity via regulating oxidative stress-activated MAPK and Nrf2 signaling pathways in the exposed Nile tilapia. Additionally, phytochemicals exhibit anti-inflammatory properties, which further help in mitigating the oxidative damage caused by pesticides in fish [23]. By inhibiting pro-inflammatory cytokines and suppressing the NF-κB signaling pathway, these compounds reduce the inflammation and tissue damage commonly associated with pesticide exposure [234, 235]. This complementary anti-inflammatory mechanism aids in preserving cellular integrity and maintaining the overall health of fish in contaminated environments.

### 4.2. Detoxification and Biotransformation

The detoxification of pesticides in fish occurs primarily through a series of enzymatic processes categorized into three phases: Phase I (functionalization), Phase II (conjugation), and Phase III (excretion) [236, 237]. Phytochemicals have been shown to influence all three phases, enhancing the capacity of fish to metabolize and eliminate pesticides more efficiently [238]. By modulating these detoxification pathways, plant-derived compounds help reduce the accumulation of toxic pesticide residues in fish tissues, lowering the risk of long-term toxicity. Phase I detoxification involves the introduction of reactive or polar groups into hydrophobic pesticide molecules, primarily through the action of cytochrome P450 enzymes [239]. Phase II of detoxification is mediated by Phase II enzymes, including GST, UDP-glucuronosyltransferases (UGTs), and sulfotransferases (SULTs).

Phytochemicals, particularly flavonoids and terpenoids, have been shown to modulate the expression and activity of CYP enzymes [240, 241], either inducing or inhibiting their activity depending on the specific phytochemical and the context of exposure [242]. For example, polyphenols like quercetin, resveratrol, naringenin, hesperidin, and rutin inhibited CYP1A1 activity [243]. However, Křížková et al. [244] reported an increase in the activity of CYP enzymes after administration of quercetin and rutin in rat liver. In the study of Deng et al. [241], bilobalide, ginkgolide A, B, quercetin, and kaempferol induced CYP activity. Terpenoids and flavonoids from *Ginkgo biloba* extract stimulated the expression of hepatic CYP enzymes through pregnane X receptor, constitutive androstane receptor, and aryl hydrocarbon receptor-mediated pathways [240]. Curcumin and quercetin at low concentrations increased activity of Phase II enzymes of detoxification [245].

ATP-binding cassette (ABC) transporters like multidrug resistance proteins (MRPs) play a central role in the phase III (i.e., excretion) of detoxification process [246]. Phytochemicals have been also shown to influence the expression and function of these transporters, promoting the efficient excretion of pesticides [247]. Silymarin, a phytochemical derived from *Silybum marianum*, has been reported to upregulate the expression of MRP1, facilitating the expulsion of pesticide metabolites from fish hepatocytes 2021. Phytochemicals can also activate the Nrf2 signaling pathway, a critical regulator of cellular detoxification and antioxidant responses [248, 249]. Upon activation, Nrf2 translocates to the nucleus, where it binds to AREs in the promoter regions of genes involved in both Phase II detoxification and antioxidant defense. Phytochemicals such as sulforaphane and genistein have been demonstrated to activate Nrf2, leading to the induction of detoxifying enzymes like GST, UGT, and NQO1, as well as antioxidant enzymes like CAT and SOD [250].

### 4.3. Anti-Inflammatory Effects

Chronic inflammation is one of the most prominent responses to pesticide exposure in fish [251]. Pesticides, such as organophosphates, pyrethroids, and neonicotinoids, induce oxidative stress, leading to the release of pro-inflammatory cytokines and the activation of inflammatory signaling pathways in fish [31]. This inflammation, if prolonged, results in tissue damage, impaired immune function, and organ dysfunction, which can compromise fish health and survival. Recent studies have highlighted the role of phytochemicals in mitigating pesticide-induced inflammation.

One of the primary mechanisms by which phytochemicals exert anti-inflammatory effects is by reducing the production of pro-inflammatory cytokines [252–254]. Pesticide exposure in fish typically leads to the upregulation of cytokines such as TNF-α, IL-6, and IL-1β, which perpetuate the inflammatory response [255]. Phytochemicals, particularly polyphenols like quercetin and resveratrol, have been shown to suppress the expression of these cytokines, thereby attenuating inflammation in fish. For instance, quercetin downregulated the expression of inducible nitric oxide synthase (iNOS), IL-1β, IL-6 and nuclear transcription factors-3B in the common carp (*Cyprinus carpio*) cells [256]. Tannic acid inhibited ATR-induced inflamation by downregulating the expression of TNF-α, IL-1β, IL-6, and INF-γ in the grass carp hepatocytes [257]. Thymol ameloriated the deltamethrin-induced inflamation by downregulating the expression of NF-κB p65, TNF-α, IL-1β, IL-8, and IL-6 [79]. Resveratrol reduced the expression of IL-1β, IL-6, IL-8, and TNF-α in the H_2_O_2_-Nile tilapia [256].

It is recognized that pesticides activate the NF-κB activity signaling pathway, a central regulator of inflammation and immune responses in fish [79, 258]. Phytochemicals have been shown to modulate NF-κB activity, thereby reducing inflammatory responses [235]. Curcumin, a well-known anti-inflammatory phytochemical, has been found to inhibit NF-κB activation by preventing the phosphorylation and degradation of its inhibitor, IκB-α [259]. Flavonoid fraction of guava leaf extract mitigated lipopolysaccharide-induced inflammatory response via blocking of NF-κB signalling pathway in *Labeo rohita* macrophages [260]. Astaxanthin reduced the inflammation induced by lipopolysaccharide in *Channa argus* by inhibiting NF-κB and MAPKs signaling pathways [261]. Similar effect was found with quercetin, as ameloriated DEHP exposure-induced pyroptosis in grass carp, *Ctenopharyngodon idella* L8824 cell line by inhibiting ROS/MAPK/NF-κB pathway [262]. Ferulic acid showed a protective effect on oxidative stress induced by the pesticide, difenoconazole via inhibiting NF-κB pathway [263].

Cyclooxygenase-2 (COX-2) and iNOS are enzymes that play critical roles in the inflammatory response [264, 265]. Pesticide exposure in fish has been shown to upregulate the expression of COX-2 and iNOS, leading to increased levels of prostaglandins and NO, which contribute to tissue inflammation and damage [32, 41]. Phytochemicals such as epigallocatechin gallate (EGCG) and resveratrol have been reported to suppress the expression of COX-2 and iNOS, thereby reducing the production of inflammatory mediators [266, 267]. The Nrf2 pathway is best known for its role in regulating antioxidant defenses, but it also plays a key role in anti-inflammatory responses [268, 269]. Phytochemicals such as sulforaphane have been shown to activate Nrf2, leading to the suppression of NF-κB activity and the downregulation of pro-inflammatory cytokines [270].

Phytochemicals have been found to inhibit the phosphorylation and activation of MAPKs, a key mediator of inflammatory responses in fish, thereby preventing the downstream activation of inflammatory genes [261, 271]. For example, polyphenols extracted from the agri-food waste rich in tannins, chestnut (*Castanea sativa*) shell, and mullein (*Verbascum macrurum*) induced the anti-inflammatory responses in zebrafish, *Danio rerio* by modulating MAPK pathway, inhibiting p38 phosphorylation and increasing extracellular-signal-regulated kinase activation, which subsequently led to suppression of NF-kB pathway [272].

Pesticide-induced oxidative stress is a major contributor to the inflammatory response in fish [31]. Excessive ROS produced during pesticide exposure can damage cellular components and activate inflammatory signaling pathways. Phytochemicals with potent antioxidant properties, such as flavonoids and phenolic acids, can reduce oxidative stress and, consequently, inflammation in fish [23, 187].

### 4.4. Chelation of Pesticide Residues

Some pesticides, including fungicides and insecticides, contain toxic metals such as copper, cadmium, and lead [273, 274], which are known to accumulate in fish tissues and cause oxidative stress, enzyme inhibition, and cellular damage [275–277]. Heavy metals present in these pesticides, such as cadmium and lead, can disrupt the normal function of detoxification enzymes in fish, such as GST and CAT, by binding to their active sites [43]. Phytochemicals such as polyphenols, flavonoids, and tannins possess multiple hydroxyl groups and aromatic rings, which allow them to bind metal ions, forming stable complexes that reduce the bioavailability and toxicity of the metals [278–282]. For instance, quercetin has been shown to chelate metal ions like lead in Nile tilapia, preventing the formation of ROS and subsequent oxidative stress [283]. By chelating Pb, curcumin decreased the metal accumulation in tissues and increased the survival of Pb-exposed common carp [284]. Polyphenols like epigallocatechin gallate (EGCG) could chelate metal ions, thus preventing their interaction with key enzymes [285]. In the study of Hussain et al. [286], phenolics and flavonoid compounds chelated and removed pesticide residues. In addition, It is known that flavonoids have the ability to chelate free radicals generated by oxidative stress [287, 288], although a study it was not studied in fish with pesticide-induced oxidative stress. In conclusion, metal-containing pesticides and their residues can accumulate in fish tissues, leading to oxidative stress and enzyme inhibition. Phytochemicals, including polyphenols, flavonoids, and tannins, have demonstrated the ability to chelate metal ions, reducing their bioavailability and toxicity. The chelating properties of phytochemicals offer a promising approach for mitigating the harmful effects of pesticide-related metal exposure in aquatic organisms.

### 4.5. Improved Nutritional Status

Exposure to pesticides such as organophosphates, carbamates, and pyrethroids disrupts nutrient absorption, metabolism, and energy utilization in fish, leading to malnutrition, growth retardation, and compromised health [289, 290]. Phytochemicals have emerged as a potential solution to ameliorate the adverse effects of pesticide exposure by enhancing the nutritional status of fish [291]. Pesticide exposure can impair the activity of digestive enzymes in fish, leading to reduced nutrient absorption and compromised growth [290, 292, 293]. Phytochemicals, particularly those with antioxidant properties such as quercetin, turmeric, ginger, and garlic have been shown to enhance the activity of these enzymes, thereby improving nutrient absorption in fish [294, 295]. By protecting and restoring enzyme function, phytochemicals help improve the digestive efficiency of fish under pesticide stress.

The gut microbiota plays a crucial role in nutrient metabolism and overall health in fish [296]. Pesticides can disrupt the balance of gut microbiota, leading to dysbiosis, reduced nutrient absorption, and impaired immune function [297, 298]. Phytochemicals can positively influence gut health by promoting the growth of beneficial bacteria and suppressing harmful microorganisms [299]. There are limited studies on the effects of phytochemicals on the gut microbiota in fish. For instance, Giannenas et al. [300] observed a higher intestinal population of *Lactobacillus Spp*. in the rainbow trout, *Oncorhynchus mykiss*, after supplementation of the fish with thymol, and carvacrol. Quercetin improved the stability of probiotic bacteria, *Lactobacillus* and *Bacillus* spp in the intestinal flora of Dark Sleeper, *Odontobutis potamophila* [301]. This improvement in gut health and microbiota composition may help counteract the negative impact of pesticides on fish nutrition.

Phytochemicals can also enhance the metabolic capacity of fish by stimulating key metabolic pathways that are often disrupted by pesticide exposure [291]. Phytochemicals such as flavonoids, saponins, and carotenoids have been shown to activate enzymes involved in carbohydrate, lipid, and protein metabolism. For example, gallic acid, theaflavin, and glycoprotein could activate enzymes in the tricarboxylic acid (TCA) cycle [282, 302]. The phytogenic supplement containing olive by-product and green tea extracts could also enhance protein synthesis in Largemouth Bass, *Micropterus salmoides* by activating key signaling pathways such as the AKT-mTOR pathway, which regulates cell growth and protein production [303].

Additionally, phytochemicals as growth promotor can improve the bioavailability of amino acids [291], ensuring that fish have sufficient building blocks for protein synthesis, even in the presence of pesticides. For example, the use of chitosan in the diet of stellate sturgeon (*Acipenser stellatus*) juveniles improved the fish growth through developing gut morphology and increasing nutrient absorption capacity [304].

Pesticide-induced oxidative stress not only compromises cellular integrity but also reduces the availability of lipids for energy production and membrane synthesis [277, 305, 306]. Phytochemicals with strong antioxidant properties, such as carotenoids, tocopherols, and flavonoids, can reduce lipid peroxidation and protect essential fatty acids from oxidative damage [307, 308]. By preserving the integrity of lipids, phytochemicals help maintain healthy cell membranes and ensure a steady supply of energy from fat stores, thereby improving the nutritional status of pesticide-exposed fish [309]. Furthermore, the effect of phytochemicals on fish growth may be managed through modulation of the growth hormone axis. The addition of curcumin (0.5%) in the diet of tilapia, *Oreochromis mossambicus* elevated the expression of growth hormone in brain and also enhanced mRNA level of IGF-1 and IGF-II in liver [310]. Similarly, D-limonene at a dietary level of 4 and 6 mg/kg in the diet of tilapia stimulated the expression of growth hormone and IGF-I [311].

Heavy metals in the structure of metal-containing pesticides can interfere with the absorption of essential nutrients by competing for binding sites in the fish gastrointestinal tract [312, 313]. These toxic metals not only disrupt nutrient uptake but also accumulate in tissues, leading to further toxicity [314]. Phytochemicals with chelating properties, such as tannins, phenolic acids, and flavonoids, can bind to heavy metals and facilitate their excretion from the body [278, 281, 282].

Oxidative stress caused by pesticide exposure can degrade vital nutrients such as vitamins, lipids, and proteins in fish [38, 105, 315]. Phytochemicals, known for their antioxidant properties, can protect these nutrients from oxidative damage. For example, vitamins A, C, and E, which are susceptible to degradation by ROS, can be preserved by the action of antioxidant phytochemicals like polyphenols and carotenoids in fish [187, 316]. Phytochemicals such as resveratrol, curcumin, and flavonoids enhance the endogenous antioxidant defense system [216], thereby preserving vital nutrients for fish growth.

In conclusion, pesticide exposure in fish significantly disrupts nutrient absorption, metabolism, and overall health, leading to growth impairments and oxidative stress. Phytochemicals have emerged as a promising solution to counter these adverse effects by enhancing digestive enzyme activity, promoting nutrient absorption, and protecting cellular integrity. Additionally, they help restore gut microbiota balance, chelate toxic metals, and mitigate oxidative damage, improving fish growth and health. Through antioxidant properties and modulation of key metabolic pathways, phytochemicals offer a natural, effective approach to improve fish resilience against pesticide-induced stress.

### 4.6. Immunomodulation

Pesticide exposure in aquatic environments compromises the immune system of fish, making them vulnerable to infections, diseases, and reduced survival rates [27, 295].

Phytochemicals have been shown to enhance the function of the immune cells (i.e., macrophages, neutrophils, and lymphocytes), restoring their ability to fight infections in pesticide-exposed fish [214]. For instance, flavonoids like quercetin and curcumin have been reported to boost the phagocytic activity of macrophages and stimulate the production of ROS necessary for pathogen destruction [317]. Also, flavonoids like quercetin and curcumin have been reported to boost the phagocytic activity of macrophages and stimulate the production of ROS necessary for pathogen destruction [317]. Many researches have studied the effects of phytochemicals on fish immune parameters.  Table 2 presents a selection of these studies. However, the mechanisms involved in this function are much less well-known in fish.

Pesticides also can disrupt the balance of pro-inflammatory and anti-inflammatory cytokines, leading to chronic inflammation or immune suppression in fish [255, 323]. Phytochemicals are known to modulate inflammatory and anti- inflammatory by cytokine and inflammatory mediator production, restoring immune balance and improving immune defense mechanisms. For example, resveratrol ameliorates the inflammatory response by down-regulating the IL-1β, IL-6, IL-8, and TNF-α in the common carp [324]. Similar results were observed in the red tilapia (*O. mossambicus*♀× *O. niloticus*♂) [325], in gibel carp, *Carassius gibelio* [326] supplemented with resveratrol. Quercetin downregulated the expression of iNOS, IL-1β, IL-6 and nuclear transcription factors-3B in the common carp (*C. carpio*) cells [256]. Tannic acid inhibited ATR-induced inflammation by downregulating the expression of TNF-α, IL-1β, IL-6 and INF-γ in the grass carp hepatocytes [257]. Thymol ameliorated the deltamethrin-induced inflammation by downregulating the expression of NF-κB p65, TNF-α, IL-1β, IL-8 and IL-6 [79]. Phytochemicals also can regulate anti-inflammatory responses by depressing the production of inflammatory mediators, including prostaglandins and nitric oxide (NO) [254]. The phytochemical-induced reductions in prostaglandins are mediated through inhibiting the activity of the enzyme, cyclooxygenase-2 (COX-2), which is responsible for prostaglandin synthesis [327]. For example, quercetin exerts a neuroprotective effect by inhibiting the iNOS/NO system and the expression of pro-inflammation genes in PC12 cells in zebrafish [328].

Phytochemicals can influence immune function at the molecular level by regulating the expression of genes involved in coding immune receptors, cytokines, and antimicrobial peptides. Phytochemicals, particularly polyphenols, can activate transcription factors like NF-κB and activator protein-1 (AP-1), which are crucial for the expression of immune-related genes [329–332]. For instance, chitosan and chitooligosaccharides attenuate intestinal inflammation in the turbot, *Scophthalmus maximus* by modulating NF-кB, AP-1 and MAPKs pathways [333].

Phytochemicals have demonstrated the ability to protect immune cells from apoptosis, thus preventing immune suppression. Many phytochemicals, such as flavonoids, carotenoids, and curcumin, possess strong antioxidant properties that help neutralize ROS [216]. ROS are byproducts of cellular metabolism that can induce oxidative stress, which is a major trigger for apoptosis in immune cells [334]. Phytochemicals also play a role in modulating key signaling pathways that regulate apoptosis, such as the PI3K/Akt and MAPK pathways [335, 336]. The PI3K/Akt pathway, in particular, promotes cell survival and inhibits apoptotic signals [337, 338]. Chronic inflammation can induce apoptosis in immune cells [339], further weakening the immune system. Quercetin is antiapoptotic by blocking the mitochondrial apoptotic pathways in fish lymphocytes [308]. The phytochemical, epigallocatechin-3-gallate could protect the normal immunity of fish by inhibiting nodularin-induced apoptosis by regulating bax/bcl-2 pathway and blocking the mitochondrial apoptosis pathways with increased intracellular antioxidant enzyme activity [340].

Immune cells are particularly vulnerable to oxidative stress induced by pesticide exposure, which can impair their function and survival [173]. Phytochemicals with strong antioxidant properties, such as flavonoids, tannins, and carotenoids, protect immune cells from oxidative damage by scavenging ROS and enhancing the activity of endogenous antioxidant enzymes like SOD and CAT [216]. This antioxidant protection is critical for preserving immune cell integrity and function. The antioxidant properties of phytochemicals such as quercetin, curcumin, thymol, and resveratol have been reported in various studies in fish, which can highlight their role in cells, including immune cells against oxidative stress [79, 225, 341, 342].

In conclusion, pesticide exposure severely compromises the immune system of fish, making them vulnerable to diseases and inflammation. Phytochemicals have shown promise in restoring immune function by enhancing macrophage and neutrophil activity, regulating cytokine production, and modulating inflammatory pathways. These compounds, such as quercetin, curcumin, and resveratrol, help balance pro- and anti-inflammatory responses while protecting immune cells from oxidative damage and apoptosis. Through their antioxidant and anti-inflammatory properties, phytochemicals not only preserve immune cell integrity but also improve fish resilience against pesticide-induced immune suppression, highlighting their potential in aquaculture practices. Also, it should be stated that given the toxicity of some phytochemicals at high doses for fish, it is necessary to specifically study the appropriate dosage for target species. This issue along with testing the synergistic effects of phytochemicals optimizing phytochemical delivery methods and enhancing their bioavailability—such as using niosome carriers—can be two key areas to focus on in future studies.

## Figures and Tables

**Table 1:** The effects of some common pesticides on immune indices of some representative fish.

Pesticide	Concentration	Exposed fish	Immune components	Ref.
Chlorpyrifos	149 μg L−1 µg/L	*Cyprinus carpio*	↓ Lysozyme activity, ↓ IgM content	Li et al. [159]
Cypermethrin	0.1 µg/L	*Oncorhynchus mykiss*	↓ Total Ig, ↓ White blood cells	Cakir et al. [160]
Malathion	0.24 mg/L	*Oncorhynchus mykiss*	↓ Respiratory burst and lysozyme and complement activity, ↓ Total Ig	Hajirezaee et al. [161]
Glyphosate	104.15 mg/L	*Oncorhynchus mykiss*	↓ Phagocytic activity ↓	Le Du-Carrée et al. [162]
Atrazine	1.02 mg/L	*Rhamdiaquelen*	↓ Phagocytic and bactericidal and peroxidase and lysozyme activity	Kreutz et al. [163]
Endosulfan	4–7 µg/L	*Oreochromis niloticus*	↓ Phagocytic activity	Girón-Pérez et al. [164]
Fenvalerate	3 µg/L	*Gobiocyprisrarus*	↑ Neutrophil count, ↓ Alkaline phosphatase and lysozyme and complement activity, ↓ Total Ig	Zhang et al. [165]
Diazinon	0.39–78 mg/L	*Oreochromis niloticus*	↑ Respiratory burs activity, ↑ Total Ig	Girón-Pérez et al. [166]
Paraquat	48.2 mg/L	*Oncorhynchus mykiss*	↓ Lysozyme and complement activity, ↓ Total Ig	Tukmechi et al. [167]

**Table 2:** The effects of some most common representative phytochemicals on immune and antioxidant responses of some representative fish and possible mechanisms involved.

Phytochemical	Dietary level	Fish	Ref.	Results	Possible mechanisms
Curcumin	200–400 mg/kg diet	*Oreochromis niloticus*	Amer et al. [318]	↑ Catalase and GSH activity, ↑ ↑ Complement activity, ↑ Total IgM, ↑Immune expression of proinflammatory cytokine	Acts as an antioxidant, Activation of Nrf2 signaling pathway, reduction of oxidative stress in immune cells, cytokine production [319, 320]

Quercetin	1 g/kg diet	*Cyprinus carpio*	Jasim et al. [221]	↑ SOD, CAT, and GPx activity, ↑ Complement activity, ↑ Total IgM, ↑lysozyme, and bactericidal activity, ↑ Expression of the immune-related genes (*C3*, *Lyz*, *I*gM)	Increasing phagocytic activity, activation of antioxidant enzymes, increases in CYP activity in liver, antiapoptotic effects on fish lymphocytes, reduction of oxidative stress in immune cells, Acts as an antioxidant, modulates cytokineproduction, chelating metal containing pesticides [220, 283, 308, 317]

Resveratrol	75–125 mg/kg	*Channa argus*	Tian et al. [321]	↑ SOD, CAT, and GST, ↑ Complement activity, ↑ Total IgM, ↑lysozyme activity	Activation of antioxidant enzymes, increases in CYP activity in liver, cytokine production, inhibition of cyclooxygenase-2 (COX-2) and inducible nitric oxide synthase (iNOS) activity [125, 266, 267]

Thymol	1.5–2.5 g/kg	*Oncorhynchus mykiss*	Hafsan et al. [226]	↑ SOD, CAT, and GST, ↑ Complement activity, ↑ Total IgM, ↑lysozyme & bactericidal activity, ↑ Expression of the immune-related genes (*C3*, *Lyz*, *IgM*), ↓ Expression of the inflammatory genes	Contains epigallocatechin gallate (EGCG), which reduces oxidative stress and improves immunity, Activation of antioxidant enzymes, modulates cytokine production, Activation of JAK/STAT signaling pathway [79, 225, 322]

## Data Availability

All data generated or analyzed during this study were included in this published article.
